# *Schistosoma mansoni* and HIV infection in a Ugandan population with high HIV and helminth prevalence

**DOI:** 10.1111/tmi.12545

**Published:** 2015-06-01

**Authors:** Richard E. Sanya, Lawrence Muhangi, Margaret Nampijja, Victoria Nannozi, Prossy Kabuubi Nakawungu, Elson Abayo, Emily L. Webb, Alison M. Elliott, the LaVIISWA study team, Margaret Nampijja, Richard Sanya, Barbara Nerima, Emily Webb, Lawrence Muhangi, Beatrice Mirembe, Justin Okello, Jonathan Levin, Milly Namutebi, Christopher Zziwa, Esther Nakazibwe, Josephine Tumusiime, Carol Ninsiima, Grace Kamukama, Susan Amongin, Mirriam Akello, Robert Kizindo, Moses Sewankambo, Denis Nsubuga, Prossy Kabubi Nakawungu, Emmanuel Niwagaba, Gloria Oduru, Grace Kabami, Elson Abayo, Joyce Kabagenyi, Gyaviira Nkurunungi, Fred Muwonge, Dennison Kizito, Stephen Cose, David Abiriga, Victoria Nannozi, Bonaventure Mbaraga, Rebecca Musoke, Wambi George, James Kaweesa, Edridah Tukahebwa, Alison Elliott

**Affiliations:** ^1^ Medical Research Council/UVRI Uganda Research Unit on AIDSEntebbeUganda; ^2^ Makerere University Joint AIDS ProgrammeKampalaUganda; ^3^ Entebbe General HospitalEntebbeUganda; ^4^ London School of Hygiene & Tropical MedicineLondonUK

**Keywords:** *Schistosoma mansoni*, schistosomiasis, Bilharzia, HIV, *Schistosoma mansoni*, schistosomiase, bilharziose, VIH

## Abstract

**Objectives:**

Recent reports suggest that *Schistosoma* infection may increase the risk of acquiring human immunodeficiency virus (HIV). We used data from a large cross‐sectional study to investigate whether *Schistosoma mansoni* infection is associated with increased HIV prevalence.

**Methods:**

We conducted a household survey of residents in island fishing communities in Mukono district, Uganda, between October 2012 and July 2013. HIV status was assessed using rapid test kits. Kato‐Katz (KK) stool tests and urine‐circulating cathodic antigen (CCA) were used to test for *Schistosoma* infection. Multivariable logistic regression, allowing for the survey design, was used to investigate the association between *S. mansoni* infection and HIV infection.

**Results:**

Data from 1412 participants aged 13 years and older were analysed (mean age 30.3 years, 45% female). The prevalence of HIV was 17.3%. Using the stool Kato‐Katz technique on a single sample, *S. mansoni* infection was detected in 57.2% (719/1257) of participants; urine CCA was positive in 73.8% (478/650) of those tested. *S. mansoni* infection was not associated with HIV infection. [KK (aOR = 1.04; 95% CI: 0.74–1.47, *P* = 0.81), CCA (aOR = 1.53; 95% CI: 0.78–3.00, *P* = 0.19)]. The median *S. mansoni* egg count per gram was lower in the HIV‐positive participants (*P* = 0.005).

**Conclusions:**

These results add to the evidence that *S. mansoni* has little effect on HIV transmission, but may influence egg excretion.

## Abstract (fr)

**Objectifs:**

Des rapports récents suggèrent que l'infection à *Schistosoma* pourrait augmenter le risque de contracter le virus de l'immunodéficience humaine (VIH). Nous avons utilisé les données d'une vaste étude transversale afin de déterminer si l'infection à *Schistosoma mansoni* était associée à une prévalence accrue du VIH.

**Méthodes:**

Nous avons mené une enquête auprès des ménages de résidents dans les communautés de pêcheurs de l’île du district de Mukono, en Ouganda, entre octobre 2012 et juillet 2013. Le statut VIH a été évalué en utilisant des kits de tests rapides. La technique Kato‐Katz (KK) sur les selles et l'antigène cathodique circulant (ACC) de l'urine ont été utilisés pour déterminer l'infection *Schistosoma*. Une régression logistique multivariée, permettant la conception de l'enquête, a été utilisée pour étudier l'association entre l'infection à *S. mansoni* et l'infection par le VIH.

**Résultats:**

Les données de 1412 participants âgés de 13 ans et plus ont été analysées (âge moyen: 30,3 ans, 45% de femmes). La prévalence du VIH était de 17,3%. En utilisant la technique de Kato Katz sur un seul échantillon de selles, l'infection à *S. mansoni* a été détectée chez 57,2% (719/1257) des participants; l’ACC de l'urine était positif chez 73,8% (478/650) des personnes testées. L'infection par *S. mansoni* n'a pas été associée à l'infection par le VIH. [KK (ORa = 1,04; IC95%: 0,74 à 1,47; P = 0,81), ACC (ORa = 1,53; IC95%: 0,78 à 3,00; p = 0,19)]. Le nombre médian d’œufs de *S. mansoni* par gramme était plus faible chez les participants VIH positifs (p = 0,005).

**Conclusions:**

Ces résultats ajoutent à la preuve que *S. mansoni* a peu d'effet sur la transmission du VIH, mais l'excrétion des œufs pourrait être influencée.

## Abstract (es)

**Objetivos:**

Informes recientes sugieren que la infección por *Schistosoma* podría aumentar el riesgo de adquirir el virus de la inmunodeficiencia humana (VIH). Hemos utilizado datos de un gran estudio crosseccional para investigar si la infección por *Schistosoma mansoni* está asociada con una prevalencia aumentada de VIH.

**Métodos:**

Hemos conducido un estudio en hogares de residentes en comunidades de pescadores en el distrito de Mukono, Uganda, entre Octubre 2012 y Julio 2013. El estatus de VIH se determinó utilizando pruebas de diagnóstico rápido. La infección por esquistosoma se determinó mediante la técnica de Kato‐Katz (KK) en muestras de heces y la detección del antígeno catódico circulante (ACC) en orina. Se utilizó la regresión logística multinomial, teniendo en cuenta el diseño del estudio, para investigar la asociación entre la infección por *S. mansoni* y la infección por VIH.

**Resultados:**

Se analizaron datos de 1,412 participantes con 13 años o más (edad media 30.3 años, 45% mujeres). La prevalencia de VIH era del 17.3%. Utilizando la técnica de Kato Katz en una única muestra de heces, se detectó infección por *S. mansoni* en un 57.2% (719/1,257) de los participantes; el ACC en orina era positivo en un 73.8% (478/650) de todos aquellos a los que se les realizó la prueba. La infección por *S.mansoni* no estaba asociada con la infección por VIH. [KK (aOR=1.04; IC 95%: 0.74‐1.47, P=0.81), CCA (aOR=1.53; IC 95%: 0.78‐3.00, p = 0.19)]. La media del conteo de huevos de *S. mansoni* por gramo era menor entre los participantes VIH positivos (p=0.005).

**Conclusiones:**

Estos resultados aportan evidencia de que *S. mansoni* tiene poco efecto sobre la transmisión del VIH, pero podría influir sobre la excreción de huevos.

## Introduction

Sub‐Saharan Africa is home to more than 90% of the world's schistosomiasis cases 1 and more than 68% of people infected with human immunodeficiency virus type 1 (HIV‐1) 2. *Schistosoma haematobium* causes urogenital schistosomiasis and poses a risk for HIV acquisition through the urogenital lesions 3, 4, 5. However, *Schistosoma mansoni* rarely causes genital lesions and there is still no consensus about its impact on HIV‐1 acquisition, with variable findings on the association between the two infections reported 6, 7, 8, 9, 10, 11, 12, 13.

*In vitro* studies have shown that patients with active schistosomiasis displayed higher cell surface densities of chemokine receptors CCR5 and CXCR4, making the cells more susceptible to HIV than those from helminth‐free individuals 14. *Schistosoma mansoni* has been shown to increase susceptibility to simian–human immunodeficiency virus (SHIV) infection in rhesus macaques after mucosal challenge by CCR5 tropic (R5) SHIV 15, and the presence of *S. mansoni* in rhesus macaques resulted in higher T‐helper (Th) 2 cell numbers and SHIV replication 16. In humans, a study in rural Tanzanian villages near Lake Victoria found that *S. mansoni* infection predicted HIV infection among reproductive age women 13.

We present findings from a study conducted among island fishing communities in Lake Victoria, Uganda. Low levels of sanitation and regular contact with water from Lake Victoria (a water body highly infested with *Biomphalaria*, the intermediate snail host of *S. mansoni*) lead to a high prevalence of *S. mansoni* in these communities. The prevalence of HIV infection is also higher than the national average among these communities, and they may play a key role in the spread of HIV and in the maintenance of the HIV infection levels in the general population 17, 18.

The main objective of this study was to determine whether there is an association between *S. mansoni* infection and HIV‐1 infection in the fishing communities of Lake Victoria. A causal association between these two infections would be of huge public health importance in terms of prevention and treatment strategies for both diseases in these communities.

## Methods

### Study area and design

This was a cross‐sectional household survey conducted in 26 fishing villages in Koome Islands, Lake Victoria, Mukono district, Uganda. The survey was carried out as the baseline for an ongoing cluster randomised trial which aims to investigate the impact of anthelminthic treatment on allergy‐related diseases (The Lake Victoria Island Intervention Study on Worms and Allergy‐related Diseases, ISRCTN 47196031). The protocol for this trial has been described 19.

### Study procedures

For each village, existing household registers were checked and updated for use as the sampling frame for the survey. An equal number of households was then selected from each village by simple random sampling. Household members of all ages (defined as those who sleep in the same house and share meals) were included in the survey.

A questionnaire collecting data on social, demographic and family characteristics was completed for every household member. Data collected included gender, age, occupation, household assets, source of water for domestic use, exposure to the lake, the type of footwear worn and history of having ever received anthelminthic treatment (including praziquantel, which is effective against schistosomiasis). Voluntary HIV counselling (pre‐ and post‐test) and testing was provided by trained and experienced counsellors through collaboration with the Makerere University Joint AIDS Programme's ‘REACH‐U’ project which had the mandate for voluntary counselling and testing in Mukono District at the time of the survey.

Stool samples were requested from all participants and midstream urine samples from a subgroup of participants (during the latter part of the survey) to test for *S. mansoni* infection. Both stool and urine samples were analysed immediately in the field.

Kato‐Katz analysis of a single stool sample with duplicate slides was performed by experienced technicians, to identify and quantify *S. mansoni* infection 20. Infection intensity was categorised using WHO cut‐offs as light [1–99 eggs per gram (epg)], moderate (100–399 epg) or heavy (≥400 epg) 21.

The urine‐circulating cathodic antigen (CCA) cassette test (Rapid Medical Diagnostics, Pretoria, South Africa) was used to detect the parasite antigen CCA which is present in all *Schistosoma* species 22. The major portion of CCA released by the adult live parasite is secreted in urine. A positive CCA test result on a midstream urine specimen indicates an active *Schistosoma* infection. The procedure for the test conformed to the manufacturer's instructions. All reagents were equilibrated to ambient temperature before commencing the assay. For each sample, one drop of urine was transferred by pipette to the circular well of the test cassette. The sample was allowed to absorb entirely into the specimen pad within the circular well and one drop of buffer added. The results were read exactly 20 min after adding the buffer to the test cassette. Any result read outside 25 min was considered invalid and the test repeated. All ‘trace’ or ‘weakly reactive’ readings were reported as positive.

HIV antibody testing was performed using Determine (Abbott Laboratories, Abbott Japan, Ltd., Tokyo, Japan), Stat‐Pak (Chembio Diagnostic Systems, Inc., Medford, USA) and Uni‐Gold HIV (Trinity Biotech, Bray, Ireland) test kits as per the manufacturers' specifications and the Uganda national HIV testing algorithm 23.

### Ethics

The study was approved by the Uganda Virus Research Institute Research and Ethics Committee, the Uganda National Council of Science and Technology and the Ethics Committee of the London School of Hygiene and Tropical Medicine. Permission to include each household was obtained from the household head (or from another adult if the household head was absent). Written informed consent was obtained from all adult household members and from parents or guardians on behalf of children. Written informed assent was also obtained from children aged 8 years and above. Individuals found to be HIV positive were referred for care to the subcounty health centre (where antiretroviral therapy is available) or to relevant mainland health services. All participants were offered treatment with praziquantel and albendazole as part of the community‐wide anthelminthic intervention programme.

### Statistical analysis

Data were entered using Microsoft Access (Microsoft Corporation, Redmond, WA, USA) and analysed using STATA version 13 (Stata Corporation, College Station, TX, USA). To allow for the survey design (clustering by village, and variable village size which meant the design was non‐self‐weighting), we used STATA survey commands to calculate linearised standard errors and apply village‐level weights to the analysis.

The analysis had three aims: firstly, to identify individual and household‐level characteristics associated with HIV infection; secondly, to identify characteristics associated with *S. mansoni* infection (as ascertained from stool samples using Kato‐Katz analysis); and finally to assess the association between *S. mansoni* and HIV infections.

Children younger than 13 years were excluded from the analysis because they were unlikely to have acquired HIV infection by sexual transmission.

Variables considered as possible risk factors for both HIV and *S. mansoni* were sex, age, occupation, tribe, number of household members, asset score, type of wall, type of roof, smoking, fuel used for cooking, source of washing/drinking water, frequency of going in the lake, footwear when outside the house and history of treatment for worms. The asset score variable was generated by adding the number of assets owned by a household from a list comprising fishing boat, car, motorbike, bicycle, television, radio, mobile phone and bed. For the assessment of characteristics associated with HIV and *S. mansoni* infection, univariable logistic regression was used to assess crude associations between each potential risk factor and each of the two outcomes. Variables that showed evidence of crude association, or for which there was prior evidence of an association in the literature, were further investigated using multivariable logistic regression. Odds ratios (OR), 95% confidence intervals (CI) and adjusted Wald test *P*‐values were calculated for each characteristic.

The association between *S. mansoni* and HIV infection was investigated using multivariable logistic regression. *S. mansoni* status as ascertained from stool using Kato‐Katz, *S. mansoni* status as ascertained from urine using CCA and *S. mansoni* infection intensity (from Kato‐Katz) were each separately considered as exposures for this analysis. Models were adjusted for variables showing any evidence of association with both HIV and *S. mansoni* infections (sex, age, occupation, asset score and history of ever having received anthelminthic treatment).

## Results

### Participant characteristics

We enrolled 2316 participants (1268 males, 1048 females). Stool and urine samples were obtained from 1996 and 917 participants, respectively. A total of 1867 participants were tested for HIV. Of these, 455 were children under 13 – of whom only five (all under 4) were HIV positive. Data analysis for the association between HIV and *S. mansoni* infection was restricted to the 1412 participants who were aged 13 years and older and who had information on HIV status.

The mean age of the participants was 30.3 years (SD, 9.5). Forty‐two per cent (42.0%; 593/1412) of the participants were female. The prevalence of HIV infection was 17.3% (244/1412). Based on Kato‐Katz analysis of a single stool sample, 57.2% (719/1257) had *S. mansoni* infection. Among the subgroup of 650 participants for whom urine CCA results were available, this test was positive in 73.5% (478/650). Ninety‐two per cent of the participants mentioned the lake as their main source of water for domestic use. Mosquito control measures ranged from nets [34.8%; (492/1412)], or use of spray or coil (18.0%; 254/1412), to no control measures (47.2%; 666/1412). Regarding building materials, only 10.3% (145/1412) of the households used iron sheets to roof their houses (most used thatch with plastic sheeting) and only 10.0% (141/1412) of the households had a toilet.

### Factors associated with HIV infection

As shown in Table 1, the prevalence of HIV infection increased with age (*P* = 0.001). Being aged 30 years or above was associated with a sixfold higher HIV prevalence. There was evidence of an association between occupation and HIV, with those working in shops, saloons, bars or restaurants having the highest HIV prevalence. Analysing asset score as a continuous variable showed some evidence of an inverse association with HIV (aOR = 0.89; 95% CI: 0.79–1.01, *P* = 0.072). In the univariable analysis, having ever received treatment for worms (with the type of treatment not specified) showed an inverse association with HIV (aOR = 0.74; 95% CI: 0.56–0.99; *P* = 0.040), but the strength of this association diminished after adjusting for potential confounders.

**Table 1:** Factors associated with HIV infection

Risk factor	aHIV positive Proportion (%)	Crude odds ratio^b^ (95% CI)	*P*‐value^c^ (trend)	Adjusted odds ratio^b^ (95% CI)	*P*‐value^c^ (trend)
Sex					
Female	113/593 (19.1)	1	0.216	1	0.077
Male	131/819 (16.0)	0.84 (0.63–1.12)		0.72 (0.50–1.04)	
Age group					
13–19	5/143 (3.5)	1	0.021 (<0.001)	1	0.030 (0.001)
20–29	83/591 (14.0)	3.92 (1.36–11.30)		3.40 (0.98–11.77)	
30–39	100/433 (23.1)	7.56 (2.07–27.59)		6.61 (1.56–28.13)	
40+	56/241 (23.2)	7.59 (2.25–25.61)		7.41 (1.80–30.55)	
Occupation					
Housewife	15/106 (14.2)	1	0.091	1	0.009
Fishing	126/727 (17.3)	1.41 (0.77–2.57)		1.38 (0.67–2.83)	
Shops, saloons	24/105 (22.9)	1.87 (0.76–4.58)		2.16 (0.88–5.29)	
Bars, restaurants	27/129 (20.9)	1.42 (0.65–3.08)		1.46 (0.63–3.38)	
Agriculture/charcoal	44/233 (18.9)	1.72 (0.84–3.52)		1.38 (0.57–3.36)	
Other	7/98 (7.1)	0.54 (0.17–1.70)		0.86 (0.22–3.38)	
Asset score		0.88 (0.78–0.99)	0.043	0.89 (0.79–1.01)	0.072
Have you ever been treated for worms?					
No	107/541 (19.8)	1	0.040	1	0.17
Yes	132/851 (15.5)	0.74 (0.56–0.99)		0.83 (0.63–1.09)	

CI, confidence interval.

a HIV is the acronym for human immunodeficiency virus.

b Odds ratios estimated using logistic regression models with linearised standard errors and weighting to allow for the survey design; adjusted odds ratio (aOR) estimated from multivariable logistic regression models that included sex, age, occupation, asset score (generated by adding number of assets that household owns) and prior treatment for worms.

c *P*‐values are from the Wald test adjusted for the survey design.

### Factors associated with *S. mansoni* infection

Being male was associated with a 2.6‐fold greater odds of *S. mansoni* infection (Table 2), while increasing age showed an inverse association with *S. mansoni* infection (trend test *P*‐value = 0.005). The odds of *S. mansoni* infection were highest among fishermen and among those with frequent lake exposure. The following showed evidence of association with lower odds of *S. mansoni* infection: use of water from a spring (as opposed to the lake); wearing sandals or closed shoes (as opposed to no shoes); and having ever received anthelminthic treatment.

**Table 2:** Factors associated with *Schistosoma mansoni* infection

Risk factor	*S. mansoni* positive Proportion (%)	Crude odds ratio^a^ (95% CI)	*P*‐value^b^ (trend)	Adjusted odds ratio^a^ (95% CI)	*P*‐value^b^ (trend)
Gender					
Female	216/537 (40.7)	1	<0.001	1	<0.001
Male	503/720 (68.7)	3.26 (2.39–4.45)		2.62 (1.88–3.65)	
Age group					
13–19	78/120 (65.7)	1	0.48 (0.14)	1	0.056 (0.007)
20–29	311/523 (59.2)	0.75 (0.39–1.44)		0.58 (0.36–0.95)	
30–39	206/383 (53.8)	0.60 (0.28–1.30)		0.38 (0.19–0.78)	
40+	123/227 (53.1)	0.59 (0.25–1.40)		0.31 (0.14–0.68)	
Occupation					
Housewife	38/97 (39.2)	1	0.001	1	0.059
Fishing	440/642 (68.5)	3.24 (1.92–5.45)		1.85 (1.00–3.44)	
Shops, saloons	41/95 (43.2)	1.30 (0.65–2.59)		0.96 (0.43–2.13)	
Bars, restaurants	38/117 (32.5)	0.63 (0.35–1.15)		0.62 (0.33–1.17)	
Agriculture/charcoal	107/209 (51.2)	1.72 (0.93–3.16)		1.01 (0.47–2.18)	
Other	47/86 (33.3)	1.24 (0.60–2.57)		0.64 (0.27–1.52)	
Asset score		0.94 (0.88–1.01)	0.11	1.05 (0.95–1.15)	0.31
Anybody smoking in house					
No	493/891 (55.0)	1	0.022	1	0.83
Yes	226/366 (62.2)	1.35 (1.05–1.75)		0.98 (0.81–1.18)	
Cooking with charcoal					
No	118/189 (67.7)	1	0.044	1	0.41
Yes	601/1068 (55.3)	0.60 (0.36–0.98)		0.80 (0.45–1.39)	
Source of water for domestic use					
Lake	671/1257 (57.8)	1	0.086	1	0.058
Spring	3/16 (16.8)	0.15 (0.03–0.66)		0.17 (0.04–0.78)	
Well	5/9 (55.5)	0.91 (0.28–2.94)		0.44 (0.14–1.43)	
Communal tap	40/74 (57.2)	0.98 (0.63–1.52)		1.10 (0.48–2.52)	
How often do you go in the lake?					
Every day	530/907 (58.4)	1	<0.001 (0.009)	1	0.005 (0.43)
Almost every day	135/206 (65.5)	1.45 (1.02–2.06)		1.66 (1.01–2.74)	
Weekly	35/74 (47.3)	0.58 (0.27–1.23)		0.82 (0.33–2.02)	
Less frequently	14/63 (22.2)	0.20 (0.11–0.38)		0.39 (0.15–1.01)	
When you are outside the house, what do you normally wear on your feet?					
Nothing	30/43 (75.0)	1	0.062	1	0.004
Sandals	644/1146 (55.8)	0.42 (0.19–0.93)		0.29 (0.15–0.57)	
Closed shoes	40/60 (65.7)	0.64 (0.25–1.74)		0.29 (0.11–0.76)	
Have you ever been treated for worms?					
No	306/472 (64.6)	1	0.011	1	0.031
Yes	399/768 (52.0)	0.61 (0.42–0.88)		0.56 (0.34–0.94)	

CI, confidence interval.

a Odds ratios estimated using logistic regression models with linearised standard errors and weighting to allow for the survey design; adjusted odds ratio (aOR) estimated from multivariable logistic regression models that included sex, age, occupation, asset score (generated by adding number of assets that household owns), tribe, smoking within the household, cooking with charcoal, footwear and prior treatment of worms.

b *P*‐values are from the Wald test adjusted for the survey design.

### Association between *S. mansoni* and HIV

As shown in Table 3, no evidence of an association between *S. mansoni* infection and HIV infection was found in this population using the Kato‐Katz method (aOR = 1.04; 95% CI: 0.74–1.47, *P* = 0.81) or using the urine CCA results (aOR = 1.53; 95% CI: 0.78–3.00, *P* = 0.19). Using *S. mansoni* egg counts in stool, there was no evidence of any association between HIV status and *S. mansoni* infection intensity.

**Table 3:** Association between *Schistosoma mansoni* and HIV

Risk factor	aHIV positive Proportion (%)	Crude odds ratio (95% CI)	*P*‐value (trend)	Adjusted odds ratio^b^ (95% CI)	*P*‐value^c^ (trend)
*S. mansoni* (tested using stool KK)					
Not infected	96/538 (17.8)	1	0.78	1	0.81
Infected	116/719 (16.1)	0.95 (0.68–1.33)		1.04 (0.74–1.47)	
*S. mansoni* intensity categories					
Uninfected	96/538 (17.8)	1	0.39 (0.22)	1	0.65 (0.67)
Light (<100 epg)	58/282 (20.6)	1.27 (0.85–1.90)		1.22 (0.87–1.70)	
Moderate (100–399 epg)	31/206 (15.0)	0.88 (0.46–1.65)		0.90 (0.42–1.90)	
Heavy (≥400 epg)	27/231 (11.7)	0.68 (0.33–1.38)		0.87 (0.39–1.91)	
Schistosoma infection (tested using urine circulating cathodic antigen)					
Negative	31/172 (18.0)	1	0.15	1	0.19
Positive	99/478 (20.7)	1.42 (0.87–2.31)		1.53 (0.78–3.00)	

CI, confidence interval.

a HIV is the acronym for human immunodeficiency virus.

b Odds ratios estimated using logistic regression models with linearised standard errors and weighting to allow for the survey design; adjusted odds ratios (aORs) estimated from multivariable logistic regression models that included sex, age, occupation, asset score (generated by adding number of assets that household owns) and prior treatment for worms.

c *P*‐values are from the Wald test adjusted for the survey design.

There was no evidence of effect modification by gender (*P*‐values for interaction = 0.27 and 0.22, based on Kato‐Katz and urine CCA results, respectively). Thus, it was not necessary to separately analyse the association between *S. mansoni* and HIV for female and male participants.

### HIV infection and *S. mansoni* egg count

Among participants with *S. mansoni* infections*,* the median *S. mansoni* eggs per gram for the HIV‐positive participants (median = 102, IQR 36–321) was lower than that for the HIV negatives (median = 180, IQR 60–660), Wilcoxon rank sum test *P*‐value = 0.005.

## Discussion

This study found no evidence of an association between *S. mansoni* infection and HIV infection among island fishing communities of Lake Victoria, Uganda. Moreover, there was no evidence of an association between HIV and *S. mansoni* infection intensity. This result, from a cross‐sectional study, accords with findings from a prospective incidence study also undertaken among island and lakeshore fishing communities 24.

An important limitation of this study is its cross‐sectional design. In their incidence study among similar communities, Ssetaala *et al*. 24 showed that *S. mansoni* infection status, defined by measurement of circulating anodic antigen (CAA) in serum, was largely stable over a period of up to 18 months between enrolment and HIV seroconversion, but still, prevalent *S. mansoni* infection status assessed in a cross‐sectional design cannot tell us, with confidence, the status at the time of HIV exposure.

The use of single stool samples to assess *S. mansoni* infection status lacks sensitivity 25 and will have resulted in misclassification (and hence underestimation of the size of any true association) in this study. Moreover, HIV infection is associated with reduced *S. mansoni* egg excretion 12, and indeed, egg counts were lower in HIV‐infected and *S. mansoni‐*infected participants in this study. This effect might obscure a positive association or lead to a spurious inverse association. However, this was mitigated by the use of urine CCA for *S. mansoni* diagnosis in a large subgroup: antigen levels in blood (and hence in urine) would be expected to be similar among HIV‐positive and HIV‐negative people infected with similar worm numbers 9. Urine CCA assays cannot distinguish between *S. haematobium* and *S. mansoni*, but parasitological studies have found *S. haematobium* to be very uncommon in Uganda 26.

Earlier cross‐sectional studies (reviewed by Brown and colleagues 27) also tended to show little evidence of a positive association between *S. mansoni* infection and HIV prevalence. However, the analysis presented here was inspired by the more recent study, conducted in the Tanzanian sector of the Lake Victoria basin, which found a positive association between *S. mansoni* and HIV infection among women 13. Our study was larger, included more *S. mansoni* and HIV‐infected people, and included both men and women, but failed to confirm the Tanzanian results. Stratification by gender showed no effect modification in our study, making it unlikely that the focus on women in the Tanzanian study explains the different findings.

*In vitro* and animal studies suggest a link between *S. mansoni* and risk of HIV infection 14, 15. One possibility that remains among human populations in highly helminth endemic communities is that immunological effects of helminth exposure persist when infections are treated or cleared 28. If this is the case, then within‐community comparisons, such as the study reported here, may fail to demonstrate an important helminth‐induced effect, by contrast to the Tanzanian study whose catchment area extended further from the lake, offering participants from a more varied exposure background. Thus, with regard to *S. mansoni*, it would be of interest to compare immunological parameters thought to increase susceptibility (such as chemokine receptor expression and Th1/Th2 cellular profiles – as discussed above) between similar populations with and without *S. mansoni* exposure – such as lakeshore villages versus inland, rural villages. Unfortunately, such studies face the ‘Catch 22’ that important exposures other than schistosomiasis may well differ between such disparate communities and confound these between‐community comparisons.

Having received anthelminthic treatment in the past was associated with a reduced risk of *S. mansoni* infection, as expected. What was unexpected was the reduced risk of HIV infection associated with anthelminthic treatment on univariate analysis. This association was weakened after adjustment for confounders but was of interest given the surprising observation in Ssetaala's study of a similar, weak association between lack of praziquantel treatment and HIV incidence.

This study addresses one aspect of the ongoing discussion about the potential association between *S. mansoni* and HIV, namely the effect of *S. mansoni* infection on HIV transmission. It does not address another important aspect of the discussion, that is the potential impact of schistosomiasis on the progression of HIV infection to AIDS.

The analysis presented in this study utilised data from the baseline survey of the LaVIISWA trial. If HIV testing and viral load estimation are included in the final survey – after 3 years of intensive *vs*. standard anthelminthic intervention – LaVIISWA may have an opportunity to investigate the impact of such treatment on HIV prevalence and replication.

## Conclusions

Together with the study by Ssetaala 24, our findings suggest that, within fishing communities, current *S. mansoni* infection is not an important risk factor for HIV infection. The possibility remains that intense exposure to *S. mansoni* infection contributes to the susceptibility of fishing communities at population level and that intervention to treat schistosomiasis may have benefits for HIV transmission in these communities. These possibilities need to be further explored.

## Appendix 1

Project leaders: Margaret Nampijja, Richard Sanya, Barbara Nerima. Statisticians and data managers: Emily Webb, Lawrence Muhangi, Beatrice Mirembe, Justin Okello, Jonathan Levin. Clinical officers: Milly Namutebi, Christopher Zziwa. Nurses: Esther Nakazibwe, Josephine Tumusiime, Carol Ninsiima, Grace Kamukama, Susan Amongin. Internal monitor: Mirriam Akello. Field workers: Robert Kizindo, Moses Sewankambo, Denis Nsubuga. Laboratory staff: Prossy Kabubi Nakawungu, Emmanuel Niwagaba, Gloria Oduru, Grace Kabami, Elson Abayo, Joyce Kabagenyi, Gyaviira Nkurunungi, Fred Muwonge, Dennison Kizito Stephen Cose. Boatman: David Abiriga. HIV counselling and testing: Victoria Nannozi, Bonaventure Mbaraga, Rebecca Musoke, Wambi George. Vector Control Programme staff: James Kaweesa, Edridah Tukahebwa. Principal investigator: Alison Elliott.
